# rTMS over bilateral inferior parietal cortex induces decrement of spatial sustained attention

**DOI:** 10.3389/fnhum.2013.00026

**Published:** 2013-02-11

**Authors:** Jeyeon Lee, Jeonghun Ku, Kiwan Han, Jinsick Park, Hyeongrae Lee, Kyung Ran Kim, Eun Lee, Masud Husain, Kang Jun Yoon, In Young Kim, Dong Pyo Jang, Sun I. Kim

**Affiliations:** ^1^Department of Biomedical Engineering, Hanyang UniversitySeoul, South Korea; ^2^Department of Biomedical Engineering, Keimyung UniversityDaegu, South Korea; ^3^Department of Rehabilitation and Assistive Technology, National Rehabilitation Center Research Institute, Yonsei University College of MedicineSeoul, South Korea; ^4^Department of Neuropsychiatry and Clinical Research Institute, Seoul National University HospitalSeoul, South Korea; ^5^Department of Neurosurgery, MEG Center, Seoul National University College of MedicineSeoul, South Korea; ^6^Department of Psychiatry, Yonsei University College of MedicineSeoul, South Korea; ^7^Institute of Neurology and Institute of Cognitive Neuroscience, University College LondonLondon, UK; ^8^St. Peter's HospitalSeoul, South Korea; ^9^Osong Medical Innovation Foundation, Medical Device Development CenterChungbuk, South Korea

**Keywords:** sustained attention, vigilance, repeated transcranial magnetic stimulation, inferior parietal lobe, spatial attention

## Abstract

Sustained attention is an essential brain function that enables a subject to maintain attention level over the time of a task. In previous work, the right inferior parietal lobe (IPL) has been reported as one of the main brain regions related to sustained attention, however, the right lateralization of vigilance/sustained attention is unclear because information about the network for sustained attention is traditionally provided by neglect patients who typically have right brain damage. Here, we investigated sustained attention by applying a virtual lesion technique, transcranial magnetic stimulation (TMS), over the left and right superior parietal lobe (SPL) and IPL. We used two different types of visual sustained attention tasks: spatial (location based) and non-spatial (feature based). When the participants performed the spatial task, repetitive TMS (rTMS) over either the right or left IPL induced a significant decrement of sustained attention causing a progressive increment of errors and response time. In contrast, participants' performance was not changed by rTMS on the non-spatial task. Also, omission errors (true negative) gradually increased with time on right and left IPL rTMS conditions, while commission errors (false positive) were relatively stable. These findings suggest that the maintenance of attention, especially in tasks regarding spatial location, is not uniquely lateralized to the right IPL, but may also involve participation of the left IPL.

## Introduction

Sustained attention or vigilance can be defined as the ability to maintain or control attention over prolonged periods of time, allowing the subject to respond to critical stimuli or to inhibit responses to irrelevant stimuli (Davies and Parasuraman, 1982; Warm, 1993). Ability to sustain attention is a vital component of visual perception involving the allocation of limited processing resources appropriately to achieve the demands of current tasks. In recent years, brain imaging studies during tasks requiring sustained attention using positron emission tomography (PET) and functional magnetic resonance imaging (fMRI) have demonstrated changes in cerebral blood flow and glucose metabolism in the ventral frontal cortex and inferior parietal lobe (IPL) suggesting their involvement (Coull et al., 1998; Adler et al., 2001; Demeter et al., 2010; Tana et al., 2010). Likewise, results from lesion studies have also pointed to the same regions as being involved in sustaining attention (Wilkins et al., 1987; Richer et al., 1993; Rueckert and Grafman, 1996, 1998), for example, in patients with tumor excisions. Studies of patients with hemineglect also provide evidence that right IPL and ventral frontal cortex are crucial regions for either sustained attention or vigilance (Hjaltason et al., 1996; Robertson et al., 1997; Samuelsson et al., 1998).

Recent experiments of Malhotra et al. differentiating spatial from non-spatial vigilance have similarly suggested that the right IPL is particularly important in maintaining vigilance involving spatial locations (Malhotra et al., 2009). Patients with lesions in the right IPL and part of the intra-parietal sulcus (IPS) showed a decrement in performance over time on a visual spatial vigilance task, whereas there was no deficit on a non-spatial task. However, further supporting research is required to assure that the right IPL is involved in spatial sustained attention because many patients who participate in lesion studies generally have damage extending across other regions besides the IPL. Also, there is no pre-existing data in the literature explaining involvement of the left parietal cortex as related to sustained attention because networks for sustained attention have traditionally been studied in neglect patients. Hemi-spatial neglect, a syndrome which often is characterized by sustained attention deficits, is typically more severe and long-lasting following right hemisphere damage, although it is still unclear whether this characteristic is only related to the impairments in visuospatial attention or also extends to sustained attention (Corbetta and Shulman, 2011; Bonato, 2012; Finke et al., 2012). De Renzi et al. suggested that right brain damaged patients described in clinical studies might have, on average, larger lesions and more severe cognitive impairments (including impairments in sustained attention) than left brain damaged patients (De Renzi, 1982). Indeed, patients with larger lesions (and more severe, concurrent cognitive impairments) were typically excluded from the group of left brain damaged patients due to the presence of severe aphasia, which is much less common following right brain damage. Thus, it is possible that deficits of sustained attention in left brain damaged patients have been underestimated by previous research.

Here, we investigated sustained attention by applying a virtual lesion technique, repetitive transcranial magnetic stimulation (rTMS), over the left and right IPL. This technique has been widely used in cognitive psychology and neuro-scientific studies because it is thought to provide a direct method for inducing temporary “virtual lesions” (Jahanshahi and Rothwell, 2000; Sack and Linden, 2003; Hung et al., 2005; Nyffeler et al., 2008). In this study, we adapted Malhotra's behavioral task scheme using Transcranial magnetic stimulation (TMS) to investigate which cortical regions contribute to sustained attention. In addition to IPL, left and right superior parietal lobe (SPL) stimulation were included to assess whether sustained attention specifically rely on IPL or is controlled by other parietal regions.

## Materials and methods

### Subjects

Sixteen healthy subjects (14 males and 2 females, 24.69 ± 2.2 years old) participated in this study. Written consent was obtained from all participants according to the Severance Hospital Institutional Review Board. Each participant was paid approximately $40 USD in compensation for their effort and time. All participants were right-handed and had normal or corrected to normal vision. They were screened to ensure that they had no history of neurological or psychiatric disorders, nor any chronic physical illnesses that might cause seizures.

### Visual sustained attention task

**Figure 1:**
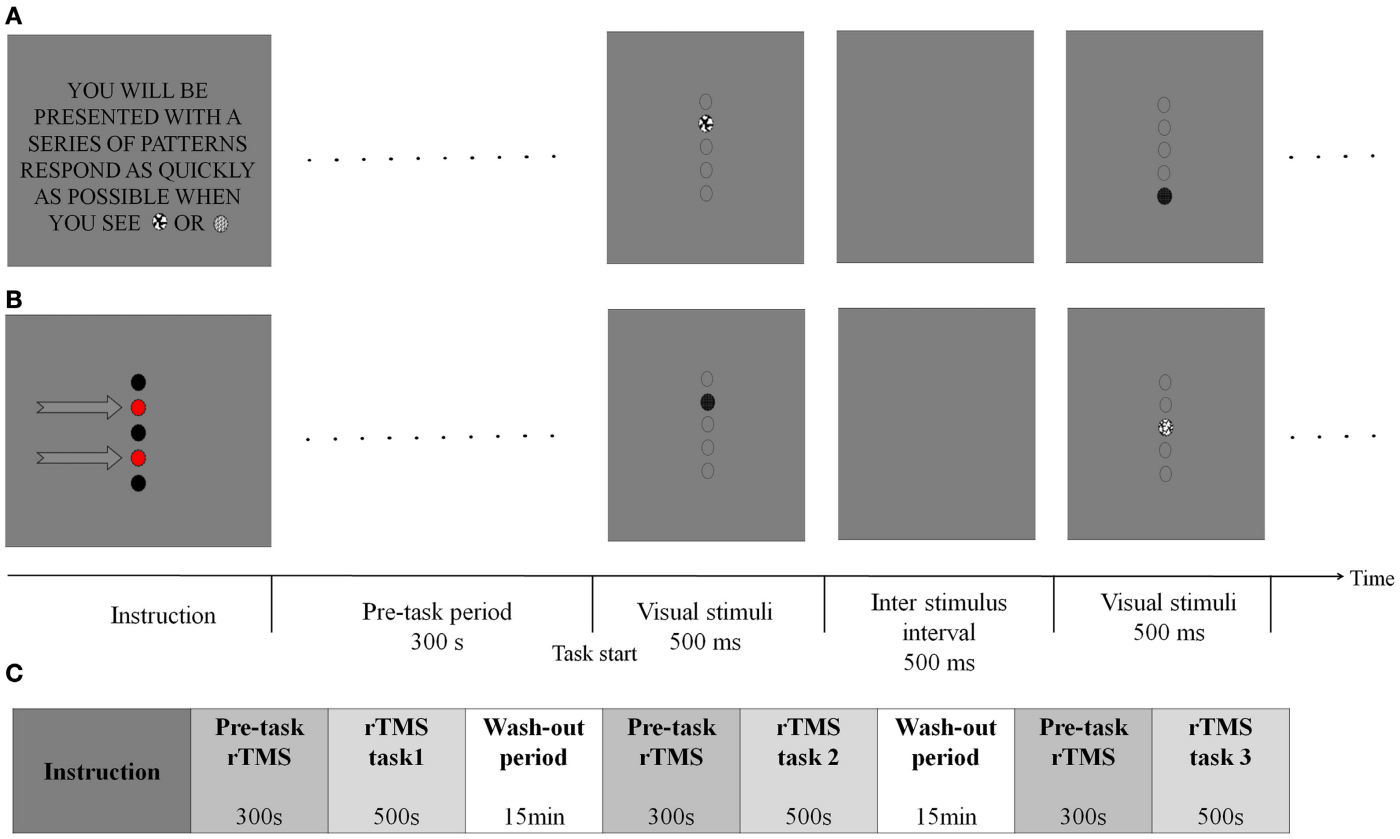
**Spatial and non-spatial sustained attention task design. (A)** In the non-spatial task, subjects were asked to respond whenever the one of two patterns was presented regardless of their spatial location. The task starts after 300 s rTMS. The first display shows a target pattern and the third display shows a non-target. Broken-line circles indicate potential target were display on a blank screen and there were no target markers. **(B)** In the spatial task, subjects were asked to respond whenever a pattern was presented at either of the two predefined locations (indicated by arrows in this figure, but not displayed during the actual experiment). The first test display shows a pattern appearing at one of the target locations. The third display shows a pattern at a non-target location. **(C)** The rTMS stimuli were applied for 5 min before behavioral task start and continued until the task finished. Total duration for rTMS stimulation was 800 s (total 800 pulses). After rTMS, 15 min was given to participants to take a rest. The order of task type (two sessions: Spatial and non-spatial task) and the order of rTMS target position (five blocks: Sham, right SPL, right IPL, left SPL and left IPL) were randomly assigned. Participants ran the two or three blocks in 1 day due to the safety issues and the remained blocks on the other day. Totally, participants took 4 days to finish the whole task.

The task combined spatial (location-based) and non-spatial (feature-based) visual sustained attention components as adopted from Malhotra's paradigm (Malhotra et al., 2009). In both tasks, circular visual stimuli were used on a uniform gray background. The visual stimuli consisted of five different patterns which were presented sequentially in random order at one of five possible locations. In the spatial task, subjects were asked to respond as quickly as possible whenever a stimulus was presented at either of the two predefined locations as indicated by red circles in Figure 1B. In contrast, in the non-spatial task, subjects were instructed to quickly respond whenever either of the two predefined target patterns of stimulus was displayed regardless of its spatial location (Figure 1A). Each stimulus remained on the screen for 500 ms and inter-stimulus intervals were also 500 ms. In total, 500 stimuli (200 targets and 300 non-targets) were presented over a period of 500 s. Subjects responded by clicking a mouse button using the index finger of the right, dominant hand.

Participants were comfortably seated while head movements were restrained for precise rTMS. Visual stimuli were presented to subjects through a head-mounted display (HMD), to minimize the potential that participants might use the edge of a video display monitor as a reference frame for making spatial decisions. Since contra-regional ignorance is a well-known symptom of patients with impairment in right PPC-hemi-spatial neglect, visual stimuli were given along the vertical median line of the screen to exclude this complication.

The task was developed using VIZARD software (World Viz Inc.). Order of task conditions and rTMS positions were randomized for each subject in order to control for order effects or maturation effects during the sustained attention test.

### rTMS

rTMS was performed over four different sites of the scalp, corresponding to the right and left SPL and IPL as determined by the following procedure. In order to precisely determine stimulation sites, each participant had a T1-weighted anatomical scans with a conventional head coil at 1.5T MRI (Signa Eclipse; GE Medical Systems) wearing a tightly fitting cap marking Fz, Cz, C3, C4, Pz, P3, P4, P7, and P8 of a 10–20 electroencephalogram (EEG) coordinate system with capsules containing soya oil. In order to determine the position of SPL and IPL in the 10–20 EEG cap, we manually extracted left and right IPS' positions in the individual T1 image and then they were mapped into 10–20 EEG system points marked by MR contrast agent. SPL position was determined 2 cm dorsal to IPS and IPL 2 cm ventral to IPS.

A custom-built, figure-of-8, TMS coil (Magstim Company Ltd.) was used for our experiment. The fixed rTMS intensity was applied at 60% of the maximum output of the stimulator's machine rather than an intensity based on the motor threshold, as the threshold in motor and non-motor cortical areas might be different (Stewart et al., 2001; Boroojerdi et al., 2002; Robertson et al., 2003; Dambeck et al., 2006). Stimulation frequency was 1 Hz because stimulation at 1 Hz has been shown to reduce blood flow and cortical excitability in the brain regions targeted by rTMS for several minutes after stimulation (Pascual-Leone et al., 1994; Chen et al., 1997; Siebner et al., 1999; Maeda et al., 2000). The TMS coil was carefully placed over the marked target site and fixed by means of a custom coil holder. As a control, sham stimulus was applied over the right IPL with a sham coil designed to be identical in appearance and clicking sound as the real coil (Magstim Company Ltd.).

We selected an online rTMS paradigm in order to eliminate the possibility of a change in the degree of virtual lesion effect as time passes (Eisenegger et al., 2008). Generally, offline approach would be more suitable for a purpose of attention task due to unspecific effects of online rTMS such as the lateralized clicking sound or the somatosensory sensation of the coil on the scalp which can cause disturbance of attention. However, we used the online approach rather than offline, because the online approach could be more effective to avoid any effect of the virtual lesion dissipates before completion of the task. The rTMS stimuli were applied for 5 min before the beginning of each behavioral task in order to induce a sufficient virtual lesion and the rTMS continued until the task finished. Total duration for rTMS stimulation was 800 s (total 800 pulses). Before subsequent application of real or sham rTMS, a 15 min rest period was provided to participants in order to remove the possibility of rTMS after-effect in a subsequent testing session. We chose the wash-out period of 15 min because a previous study showed that stimulation of the motor cortex for 10 min results in a modulation of cortical excitability that lasts for less than 10 min in healthy subjects (Romero et al., 2002).

The order of task type (two sessions: Spatial and non-spatial task) and the order of rTMS target position (five blocks: Sham, right SPL, right IPL, left SPL, and left IPL) were randomly assigned. Participants ran two or three blocks in first day and the remaining blocks on a second day in order to minimize safety issues (Rossi et al., 2008). Participants took 4 days total to finish the whole protocol (Figure 1C).

### Analysis

A sustained attention deficit is characterized by either an increase of errors or an increase in response time over time on task (Davies and Parasuraman, 1982; Matthews, 2000; Warm et al., 2008). Thus, we extracted response time, commission errors (true negative errors), omission errors (false positive errors) and total errors (commission errors + omission errors) from each task as parameters for measuring sustained attention. Responses >500 ms were classified as omissions and were used in the omissions analysis. This 500 ms criterion was adopted to better emphasize the dataset. For analysis, we divided total time into five successive periods, each consisting of 100 trials of 100 s. Each variable was averaged within each of these compartments.

In order to check whether there was a trend of significant increasing pattern of errors and response time during task with each and every rTMS condition, we performed statistical tests for the slopes of the trend with one-tailed *t*-tests. In a first step, we computed the beta value for each participant, task and stimulation site by entering the time as predictor from the beginning of the experiment and the number of errors and response time as predicted variable. In a second step, we ran one-tailed *t*-tests to test whether the regression weights of the group deviated significantly from zero. All data were analyzed using SPSS 18.0 (Chicago, IL).

## Results

Three-way repeated measures were conducted for errors and response time with task, rTMS condition, and period, and were compared using ANOVA. There was a significant effect dependent on type of task for errors [*F*_(1, 15)_ = 64.584, *p* < 0.001] and response time [*F*_(1, 15)_ = 44.569, *p* < 0.001] such that subjects made more errors and responded more slowly on the non-spatial task than the spatial task. Since the participants' overall performance was consistently better on the spatial task than the non-spatial task, it seems reasonable to interpret this as showing that the non-spatial type of task was more difficult than the spatial task.

**Figure 2:**
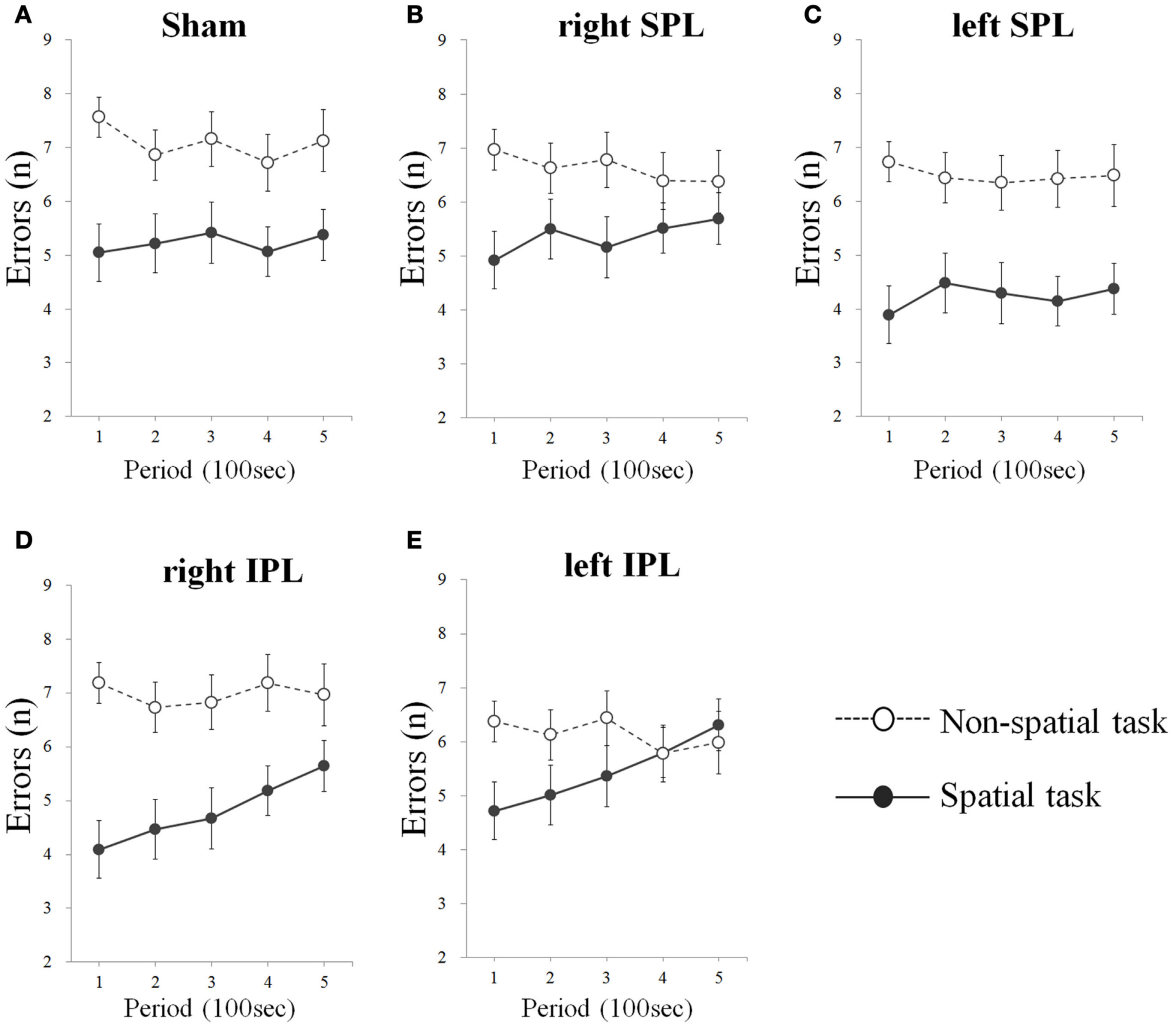
**(A–E)** Errors over time whilst performing two types of task. Errors were averaged at each period which consists of 20 tests (Error bars indicates SEM). Errors significantly increase on the spatial task when rTMS was performed on the inferior parietal lobe. As sham condition, the same clicking sound with the real coil as real TMS was performed on the right inferior parietal lobe. SPL and IPL stand for superior parietal lobe and inferior parietal lobe, respectively.

**Figure 3:**
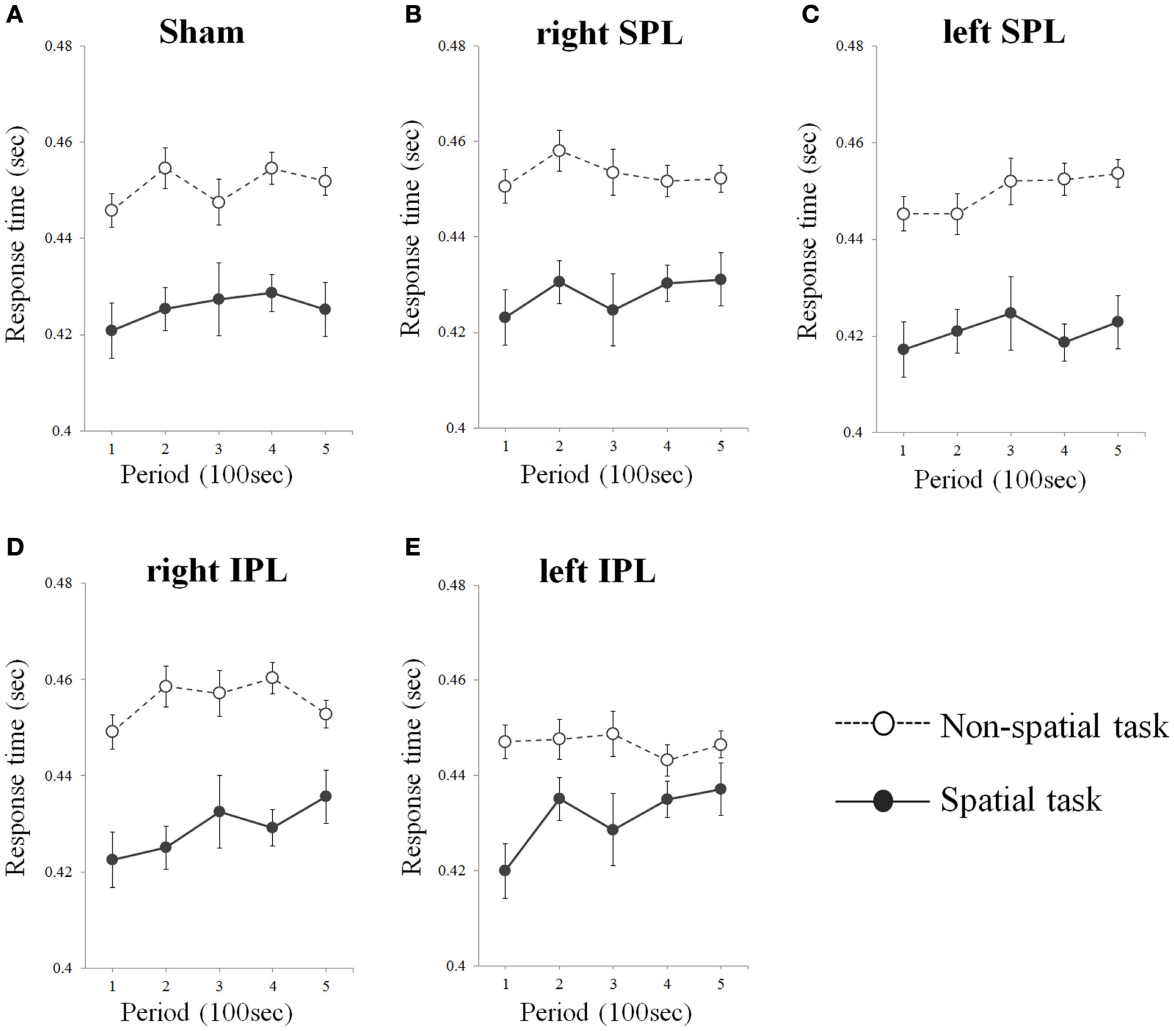
**(A–E)** Response time over time whilst performing two types of task. Response times were averaged at each period which consists of 20 tests (Error bars indicates SEM). As sham condition, the same clicking sound with the real coil as real TMS was performed on the right inferior parietal lobe. SPL and IPL stand for superior parietal lobe and inferior parietal lobe, respectively.

As a result from one-tailed *t*-test for the betas separately for every condition, significant difference of errors was found in right IPL (*t* = 3.929, *df* = 15, *p* = 0.001) and left IPL stimuli condition (*t* = 3.902, *df* = 15, *p* = 0.001) during spatial task, whereas no significant change of errors and response time was found irrespective of target area in non-spatial task (Table 1). That is, subjects showed progressive decrement of performance under right IPL and left IPL stimuli conditions in the spatial task over the course of the task (Figures 2D and E). There were increasing patterns of the response times in the spatial task under right and left IPL conditions, but not statistically significant (Figures 3D and E).

**Table 1:** **Statistical analysis of errors and response time in the spatial and non-spatial tasks**.

**rTMS target**		**Errors**		**Response time**	
		**Non-spatial task**	**Spatial task**	**Non-spatial task**	**Spatial task**
		***t*-value (*p*-value)**	***t*-value (*p*-value)**	***t*-value (*p*-value)**	***t*-value (*p*-value)**
SPL	left	−0.355 (0.728)	0.502 (0.623)	−1.269 (0.224)	0.430 (0.673)
	right	−1.511 (0.152)	1.299 (0.214)	−0.324 (0.750)	1.385 (0.186)
IPL	left	−1.169 (0.261)	**3.902 (0.001)**	−0.736 (0.473)	0.769 (0.454)
	right	−0.021 (0.983)	**3.929 (0.001)**	−0.773 (0.452)	0.237 (0.816)
Sham		−1.199 (0.249)	0.478 (0.640)	−0.817 (0.427)	0.934 (0.365)

*One-tailed t-tests for the beta values which were calculated from every participant, task, and stimulation site were performed with each and every rTMS condition. Subjects showed a significant increase in errors and a similar trend (which was not statistically significant) of increasing response time in the spatial task under right and left IPL stimuli conditions. Bold indicates P < 0.05. SPL and IPL stand for superior parietal lobe and inferior parietal lobe, respectively*.

Additionally, in order to exclude the possibility that the effects shown for right and left IPL stimulation can be alternatively explained by drowsiness or unspecific TMS effects such as lateralized click of the coil etc., we normalized the data by subtracting the errors obtained for SPL from those obtained for IPL in each individual and period for both left and right conditions (Romei et al., 2011, 2012). Then, we performed one-tailed *t*-test for beta values to confirm whether the sustained attention decrement for the spatial task survives. After normalization, significant differences were also found in right condition, i.e., right IPL minus right SPL, (*t* = 2.581, *df* = 15, *p* = 0.021) and in left condition, i.e., left IPL minus left SPL (*t* = 3.858, *df* = 15, *p* = 0.002) for only spatial task confirming that these effects are specific to IPL stimulation.

**Figure 4:**
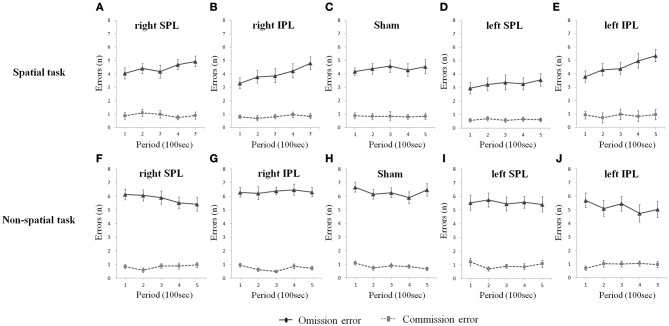
**(A–J)** Omission and commission errors over time whilst performing two types of task. Errors were averaged at each period which consists of 20 tests (Error bars indicates SEM). Only omission errors significantly increase on the spatial task when rTMS was performed on the inferior parietal lobe as time went on. As sham condition, the same clicking sound with the real coil as real TMS was performed on the right parietal lobe. SPL and IPL stand for superior parietal lobe and inferior parietal lobe, respectively.

As shown in Figure 4, omission errors (no response to stimuli, which was instructed to respond; true negative errors) and commission errors (responding to stimuli, which should not respond to; false positive errors) with the right and left IPL rTMS conditions showed different aspects by time. Omission errors increased substantially with time on the spatial task when each of the right IPL and the left IPL were disturbed [(*t* = 3.637, *df* = 15, *p* = 0.002) and (*t* = 3.262, *df* = 15, *p* = 0.005), respectively], while the level of commission errors remained relatively stable. In the non-spatial task, none of the subjects showed a change with respect to omission and commission errors.

## Discussion

In the present study, we investigated the effect of rTMS over the left and right parietal lobe using spatial and non-spatial visual sustained attention tasks. The results demonstrated that participants showed a significant decrement of performance over time of the spatial task when either the right or left IPL was disturbed by rTMS. In contrast, there was no significant change when the SPL were stimulated by rTMS regardless of side and in addition, no significant change was induced by rTMS on the non-spatial task irrespective of the target position (Figures 2A–C and 3A–C).

Notably, participant's performance on the non-spatial task was generally worse than the spatial task which demonstrates that the non-spatial task required greater effort and cognitive resources compared to the spatial task. Generally, if a task is harder and has greater demands, it often leads to a reduction in performance because vigilance tends to decline while performing highly taxing tasks. However, with IPL rTMS stimuli, performance decline was observed only on the spatial task, but not the non-spatial task, and with sham stimulation or stimulation of the SPL there was no significant decreased performance over time for either the spatial or non-spatial tasks. Therefore, the performance decrement is unlikely to be related to the taxing nature of the task. Also, the decrement was not due to a ceiling effect because it was possible for the number of errors in the non-spatial task to have increased substantially further. Thus, we can interpret these results as being due to the problem of maintaining attention on the spatial information under rTMS stimulation of the IPL rather than the task difficulty.

The result from the virtual lesion in the right IPL is consistent with previous patient studies. According to recent reports, patients with lesions in the right IPL, part of right IPS, and underlying white matter show a deficit in maintaining attention level in spatial tasks (Malhotra et al., 2009). Identically, our results show that the right IPL was responsible for spatial sustained attention. The right IPL has been previously demonstrated to be specialized for vigilance.

The most interesting point of this study is that the decrement of vigilance was observed not only with rTMS stimulation to the right IPL, but also to the left IPL where vigilance was disturbed by rTMS. There are other recent studies that likewise question the right lateralization of vigilance/sustained attention (Helton et al., 2010; Shaw et al., 2012). It seems likely that the left hemisphere is also recruited to the maintenance of vigilance with demonstrable effects dependent on increasing task difficulty and task duration. In accord with this rationale, our findings suggest that the left IPL itself does indeed play a role in the interactions involving maintenance of sustained attention with spatial information. Bonato et al. reported that a left brain damaged patient showed severe spatial deficits for the right hemispace when his attentional resources were engaged by a non-spatial and concurrent task (Bonato et al., 2010). This result makes our supposition more plausible that the left IPL are also related to the spatial sustained attentional process. Imaging studies like fMRI or PET or other rTMS studies adapting spatial and non-spatial sub-types of tasks may be able to provide further evidence. Another possible explanation of our results is that the effect of rTMS on the left hemisphere may propagate to the contra-lateral hemisphere across the corpus callosum. Several recent studies combining TMS with concurrent neuro-imaging methods have revealed that TMS also affects activity in remote regions functionally interconnected to the targeted local brain region (Bestmann et al., 2008; Blankenburg et al., 2008; Driver et al., 2009). Additionally, recent findings have shown that TMS applied to one hemisphere can have consequences for BOLD signals in the other hemisphere (Blankenburg et al., 2010). Although more supporting evidence is required, these findings suggest the possibility that rTMS in the left hemisphere also has an effect on the contra-lateral hemisphere.

In the current study, there is a significant difference between omission and commission errors within the total error increase on the spatial task. The omission errors, which are mostly due to a decrease of response rate, gradually increased as the task progressed, while commission errors remained relatively stable independent of which side of the IPL was stimulated by rTMS. This difference may be due to our adaptation of a comparatively easy task consisting of rare critical targets and relatively abundant neutral stimuli. In this task scheme, the supervisory system discriminates targets vs. neutral non-targets from successive stimuli and routinely halts motor responses to non-target stimuli while infrequently making overt responses to rare critical targets during the task because there are more neutral stimuli comparatively than targets. Therefore, if sustained attention decreases, the ability to prompt a motor response to appropriate stimuli while routinely withholding responses to the much more common non-target will decrease, and therefore true negative errors will increase, while false positive errors will decrease. Conversely, in sustained attention studies using a sustained attention to response task, commission errors were used as an indicator of vigilance decrement (Helton, 2009; Demeter et al., 2010).

One of technical points to be considered in our experiment is that the fixed rTMS intensity was applied at 60% of the maximum output of the stimulator machine. Although we adopted this protocol because the threshold in motor and non-motor cortical areas might be different, there could be a couple of issues with simply using 60% of the max stimulator output (Stewart et al., 2001; Boroojerdi et al., 2002; Robertson et al., 2003; Dambeck et al., 2006). One, it is harder to replicate this setup across devices. Two, if we were stimulating at lower amplitude and relied on a different motor threshold than what we used, we may not have seen effects at other sites or in the non-spatial task simply because we were not stimulating at a sufficient intensity.

In this study, we showed that rTMS over either the right or left IPL selectively impairs visuospatial sustained attention, but not an appropriate control task that used identical visual stimuli. Different patterns between omission and commission errors were presented. These results confirmed that the right IPL is associated with spatial sustained attention. We interpret our results as suggesting the probability that the left IPL is also involved in maintaining spatial vigilance. Further research using different designs or techniques such as combining TMS with concurrent neuroimaging would provide additional evidence to verify our findings.

### Conflict of interest statement

The authors declare that the research was conducted in the absence of any commercial or financial relationships that could be construed as a potential conflict of interest.
